# Effects of a one week multidisciplinary inpatient self-management programme for patients with fibromyalgia: a randomised controlled trial

**DOI:** 10.1186/1471-2474-13-189

**Published:** 2012-09-26

**Authors:** Bente Hamnes, Petter Mowinckel, Ingvild Kjeken, Kåre B Hagen

**Affiliations:** 1 Hospital for Rheumatic Diseases, Lillehammer, Norway; 2 National Resource Centre for Rehabilitation in Rheumatology, Department of Rheumatology, Diakonhjemmet Hospital, Oslo, Norway; 3 Department of Health Sciences, Institute of Health and Society, University of Oslo, Oslo, Norway

**Keywords:** Self-management programme, RCT, Fibromyalgia

## Abstract

**Background:**

Self-management programmes (SMP) are recommended for patients with fibromyalgia. The purpose of this study was to evaluate effects of a one week multidisciplinary inpatient self-management programme on psychological distress, skills as a consumer of health services, self-efficacy, and functional and symptomatic consequences of fibromyalgia (FM).

**Methods:**

A randomised controlled two-armed, assessor-blinded trial with three-week follow-up to evaluate SMP. Primary outcomes were the General Health Questionnaire (GHQ-20) and the Effective Musculoskeletal Consumer Scale (EC-17), while secondary outcomes included the Fibromyalgia Impact Questionnaire (FIQ) and Self-efficacy scales for pain, function and symptoms (ASES).

**Results:**

150 patients with FM were randomised to one week one SMP (n = 75) or to a waiting list control group (n = 75). Of these, 58 participants in the treatment group and 60 in the control group completed the study. At three weeks’ follow up there was a significant difference in EC-17 (0-100) in favour of the treatment group (mean difference 4.26, 95 CI 0.8 to 7.7, p = 0.02). There were no differences between the groups for any of the other outcomes.

**Conclusion:**

This study shows that in patients with FM the SMP had no effect on psychological distress, functional and symptomatic consequences and self-efficacy, except for a small short-term effect on skills and behaviour that are important for managing and participating in health care (EC-17). Clinical Trials.gov Id: NCT01035125.

**Trial registration:**

Clinical Trials.gov Id: NCT01035125

## Background

Fibromyalgia syndrome (FM) is a common rheumatic disease, with a prevalence of 3.2 in Norway. FM is characterised by chronic pain, tender points, stiffness, fatigue, depression, anxiety and sleep disturbance. FM also includes other symptoms such as headaches, migraine, abdominal pain and increased urinary frequency [1]. One review revealed that people with FM had a greater disease burden compared to people with other specific pain conditions [2].

People with FM experience a negative social response to their disease [3]. They also perceive a lack of support and acknowledgment which makes it difficult to move on in living with an invisible illness [4]. In a study among Norwegian physicians and medical students FM scored lowest on disease prestige [5]. This may affect prioritisation in medical practice.

The European League Against Rheumatism (EULAR) has developed evidence-based recommendations for management of FM. The recommendations are both pharmacological and non-pharmacological [6]. Non-pharmacological recommendations include multidisciplinary approaches, such as self-management programmes (SMP) [7-9] and cognitive behaviour therapy (CBT) [10]. Barlow defines self-management as the individual’s ability to manage the symptoms, treatment, physical and psychosocial consequences and life style changes inherent in living with a chronic condition [11]. SMPs have been developed to assist people with chronic rheumatic diseases such as FM in their efforts to cope with their disease in daily life. A review of FM studies shows that SMP with or without exercise gave no sustained benefits across a range of outcomes for more than 6 months after completion of the SMP [12].

Studies also show that the same coping intervention can vary in efficacy in patients with FM, from no improvement to significant pain reduction, increased pain-coping abilities and reduction in healthcare consumptions one year after completed intervention [13,14].

A qualitative study of patients’ expectations prior to a one week inpatient SMP revealed that they expected the SMP to be a turning point to a better future and to empower them so that they could assume more responsibility for their own health and self-care. They also expected the SMP to facilitate acceptance, help them gain new knowledge and to be a forum in which to share their experiences. Participants who were employed assumed that participation in the SMP could help to ensure that they would continue in their jobs [15].

A one-week inpatient SMP has been part of the rheumatology service at the Hospital for Rheumatic Diseases in Lillehammer for several years; therefore it was desirable to study whether it had any effects. The objective in this randomized controlled study was to evaluate the effect of a one week inpatient SMP on psychological distress, coping skills and behaviours, self-efficacy, and functional and symptomatic consequences in patients with FM.

## Methods

### Design

This was a single centre, randomised, controlled two-armed study which evaluated a one week inpatient SMP for patients with FM. The participants were allocated to the one week SMP or a waiting list control group. Participants were included at Lillehammer Hospital for Rheumatic Diseases from November 2008 to January 2010. Originally, the study was planned with follow up after three weeks and six months. However, due to an error in the study procedures, the control patients were invited to participate in the SMP before they had completed the 6-months questionnaires. Thus the 6-months follow-up data could not be collected.

### Participants

Participants were patients with FM referred for a one week SMP at Lillehammer Hospital for Rheumatic Diseases. Inclusion criteria were diagnosis of FM according to the American College of Rheumatology's criteria [16], a desire to participate in the SMP, an ability to speak the Norwegian language, age between 20 and 70 years, and willingness to give written informed consent. Exclusion criteria were previous participation in an SMP, cognitive impairment, vision or hearing problems, and serious mental disorders. The study was approved by the Regional Committee for Medical Research Ethics and the Norwegian Data Inspectorate.

Participants were assessed at inclusion before randomisation (baseline 1), three weeks before the SMP (baseline 2) and three weeks after the SMP. The time from study inclusion to participation in the SMP varied from one to six months. This was due to summer closure of the patient education unit, and postponed participation by some participants. This is the reason for the use of both baseline 1 and 2.

### Intervention

The one week inpatient SMP is a complex multidisciplinary self-management intervention [17]. The SMP has been developed on the basis of Hiim and Hippe's didactic relations model. This model contains six interrelated concepts which are central in patient education: The participant's learning abilities, pedagogical framework(s), teaching goals, contents, learning/teaching methods and evaluation. The model is developed to help teachers analyse, improve and evaluate didactic relations in practice [18,19]. The SMP programme is based on a cognitive-behavioural approach and focuses on enhancing self-efficacy and coping with the disease and daily life [20]. Self-efficacy is the belief that one will be able to perform a specific behaviour or task in the future [21]. Coping with the stress of illness involves managing the stressors (such as pain) stemming from the chronic illness [22]. The SMP also has an empowering approach aimed at enhancing participants' knowledge, attitudes, skills and behaviours and enabling them to take responsibility for their own health management and daily life [21,23-25]. The programme is described in Table 1. The inpatient education unit takes up to 16 patients and five spouses/relatives/partners per week within the same diagnostic group. Participants receive individual consultations with the multidisciplinary team if needed.

**Table 1:** The Self-Management Programme

**Day/Health profession**	**Theme**	**Goal/objectives**	**Methods**
**Sunday evening**	**Arrival**		
Nurse	Welcome and introduction	Preparation for the week.	Individual welcome
			Group introduction (1 hr)
**Monday**	**Living with chronic disease**		
Nurse	My expectations		Individual and group
	My diagnoses	Clarify expectations.	exercises, discussions
	Living with chronic disease	Awareness of the impact of the diagnoses.	Teaching and discussion
	Own goals	Introduction to important issues such as learning and normal reactions to a chronic disease. Individual targeted focus.	Writing goals (2.5 hrs)
Physiotherapist	**Exercise in swimming pool**	Learning exercises.	Group exercises (0.5 hr)
Rheumatologist, Assistant doctor	**Medical consultation**	Check of status.	Individual consultation (0.5 hr)
**Tuesday**	**Stress management**	Awareness of what triggers stress in everyday life.	Individual exercise, group discussion Teaching about stressors and stress reactions (1 hr)
Nurse			
		Understanding stress mechanisms.	
Physiotherapist	**Exercising and relaxing**	Knowledge, exercises and relaxation.	Teaching and exercises (1 hr)
Rheumatologist	**Disease and treatment**	Understanding mechanisms of the disease, what helps, and research.	TeachingDiscussions (2 hrs)
Representatives from patient organizations	**Evening Meeting**	Patient organizations as external resource.	Presentation of the organizations (1 hr)
**Wednesday**	**Self-management**	Awareness of own coping strategies, communication, values, choices. Connection between thoughts, feelings and bodily reactions.	Exercises Discussions Teaching Guided imagery (2.5 hrs)
Nurse			
Ass. Occup.Therapist	**Activity**	Tour - walking in the local area	Walking (0.5)
Occupational Therapist	**My priorities in daily life**	Awareness of how to affect own health through prioritization of daily activities and use of energy.	Teaching
			Exercise
			Discussion (1 hr)
Ass. occup.therapist	**My priorities in daily life,- daily activity**	Staying focused on positive activities.	Creative activity or visiting a museum (1.25 hrs)
**Thursday**	**Health and social welfare**	Knowledge on health and social rights, work rights.	Teaching
Social worker			Discussions (2.5 hrs)
Ass. occup.therapist	**Nordic walking**	Trying new activities.	Walking in groups (0.5 hr)
Occupational therapist	**Practical advice for everyday life**	Knowledge on ergonomics, regulation of activity, aids.	Demonstration, testing and discussions (1.25 hrs)
**Friday**	**Healthy eating**	Consciousness about own diet.	Teaching
Dietician		Knowledge on nutrition, digestion, nutritional supplements and intolerance. Promotion of positive attitudes towards healthy eating.	Discussions (2 hrs)
Nurse	**Evaluation and end of the program**	Experiences from the SMP.	Oral and written evaluation
		Focus on individual goals and the way forward.	Sharing experiences (0.5 hr)
**Monday, Tuesday, Thursday evening**	**Group session in smaller groups**	Articulating how to live with FM and how to find new coping strategies.	Discussions lead by health- care professionals (1 hr)

Before participants submitted an application for the programme, they received a brochure describing the purpose of the stay, the main topics and the teaching methods. Normal waiting time for participation in the SMP is more than a year. Participants are referred from all over the country, but the majority come from the two nearest counties. A one week SMP makes it possible for people who do not live close by to participate. All participants in the study waited a shorter time to participate in SMP than normal. The intervention group waited one to six months and the control group eight months or more from inclusion in the study until they participated in the SMP. The waiting list control group did not receive any treatment at the hospital in the period from inclusion to participation in the SMP.

### Assessments

#### Primary outcomes

The General Health Questionnaire (GHQ-20) [26-29] is a 20-item instrument for measuring psychological distress in chronic diseases. The items use a four-point Likert scale ranging from “no distress at all” to “much more distress than usual” and are summated to give a total score from 0 to 60 where 0 is the best possible score indicating no distress. A score of 24 or more is defined as pathological psychological distress [30]. Evidence of reliability and validity [28,30-32] and responsiveness in Norwegian patients [28] has been demonstrated for this instrument.

The Effective Musculoskeletal Consumer Scale (EC-17) was used to assess patient perception of skills and behaviours that are important for effectively managing, participating in or leading their health care [33].

EC-17 is a self-administered instrument with 17 items covering five skill domains: 1) How to use health information, 2) How to clarify priorities, 3) Communication with others, 4) How to negotiate own role and take control and 5) How to decide and take action. The items are summated and converted to produce a score from 0 to 100 where 100 is the best possible score [33,34]. The instrument has been translated and validated into Norwegian, and the results of a principal component analysis support the unidimensionality of the EC-17 [35].

#### Secondary outcomes

The Arthritis Self-Efficacy scale (ASES) has been developed to measure perceived self-efficacy in people with arthritis, and captures how confident an individual feels in managing symptoms such as pain, functional limitations and emotional issues. It includes three subscales for pain (5 items), functioning (9 items) and other symptoms (6 items). Each item is scored from 10 (very uncertain) to 100 (very certain). Each subscale is scored separately by taking the mean of the subscale items [36]. ASES has been used in studies of multidisciplinary self-management interventions of patients with FM [8] and measures different skills and attributes to those of the EC-17 [34].

The questionnaire included the Fibromyalgia Impact Questionnaire (FIQ) which was developed to measure the health status and the components that are believed to be most affected by FM [37]. FIQ is a self-administered questionnaire with 10 items, each of which scores 0 to 10. The score for the first item is the mean value of 10 sub-items about physical function. The second item is the number of days “feeling good” and the third item is absence from work. This question has the same modification as the validated Swedish version, where the question on absence from work has been changed from days to percentage [38]. The last seven items are visual analogue scales (VAS) for job ability, pain, fatigue, morning tiredness, stiffness, anxiety and depression. The final score is the total score of the 10 items, from 0 to 100. The average FM patient scores about 50 and the severely affected patient scores 70 or more [39].

The participants completed the first questionnaire when they were included (Baseline 1), before randomisation. The second questionnaire was returned by the participants three weeks before the programme (Baseline 2). The follow-up questionnaire was completed three weeks after the programme. All questionnaires were sent out by mail. If the participants had difficulty with completing the questionnaire, they could call the project leader.

#### Additional variables

Demographic variables included age, sex, marital status, years of education, employment status and disease duration (time since diagnosis).

#### Sample size

Power calculation was performed on the basis of a GHQ-20 included in a pilot study conducted at the hospital [35]. Based on data from 21 participants with mean 24.1 and SD 11.5 we calculated that a sample size of 73 patients for each group was required. This would detect a difference of 6 points on a scale from 0 to 60 points, with a significance level of 0.05 and a power of 80, and an expected 20 loss to follow-up.

#### Randomisation

Participants were approved for SMP by the Department’s Nursing Director and were then sent invitation to participate in the study. Participants who were included in the study were given ID numbers consecutively from 1-150 and received the first baseline questionnaire. After Baseline 1 assessments, all included participants were randomly allocated to either the treatment group or the control group. Three participants withdrew or were lost to follow-up after Baseline 1 assessments. An independent Director of Economy generated a randomised list, using Excel. An independent non-medical research assistant thereafter linked the ID number with the randomised list number. The participants were informed about group allocation after the randomization. The researcher did not have access to any data before data collection was completed.

#### Statistical methods

Frequency distribution, means and standard deviation (SD) were calculated for the demographic data at baseline for control and intervention groups. Demographic data were compared using chi-square tests for categorical variables and a t-test for continuous data.

A multivariate random effects model was applied to assess the association between the effect of the intervention on the selected primary and secondary variables at three-weeks follow up. In order to adjust for baseline values and to avoid regression to the mean, the baseline scores were included as a covariate. We calculated the ICC coefficient, version 2.1, in the Fleiss terminology, using a two-way mixed model approach [40].

In addition we adjusted for gender, education, marital status and currently employed (Yes/No). Significant variables only were included in the final model. To examine perturbation, we used Cook’s d and multivariate DfFits statistics as well as Covtracve and Covratio statistics [41]. All p-values equal to or below 0.05 (5) were considered significant. The treatment effect sizes (Cohen’s d) were calculated as the adjusted between-group difference in scores divided by the pooled SD of the baseline scores for each variable. The analysis was performed in Statistical Analysis System version 9.1.3 [42].

## Results

### Characteristics of participants

**Figure 1:**
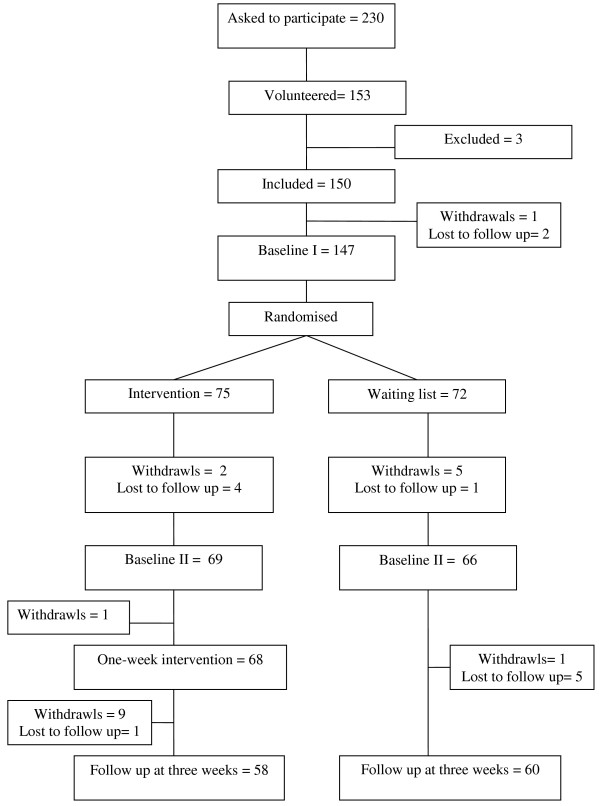
Flow diagram of the participants in the randomised trial.

Of the 230 patients on the waiting list for SMP who were asked to participate in the study, 77 (71 women and 6 men) did not return the questionnaire, (see flow diagram in Figure 1). Of the 153 patients who agreed to participate three were excluded because they had attended another SMP at the hospital. Of the 150 participants who were included in the study one withdrew and two were lost to follow up. Of the remaining 147 participants, comprising, 75 in the intervention group and 72 in the waiting list group, 118 completed the three-week follow-up, 58 (77) in the intervention group and 60 (80) in the control group.

Baseline 1 and 2 scores for the FIQ were about 60 in both groups, and lay between the mean average (50) and severely affected (70) by FM [39]. There were significant differences in gender between the two groups, with six men (8) in the SMP group, and none in the control group (p = 0.01). There were no differences in age, disease duration or other socio-demographic variables between the intervention and control group (Table 2). There were no significant correlations between the demographic variables and the total score for GHQ20, EC-17, FIQ and SEAS.

**Table 2:** **Socio-demographic characteristics for patients with FM**

**Characteristics**	**SMP group No. (%)**	**Control group No. (%)**
Sex		
Male	6 (8)	
Female	69 (92)	72 (100)
Age (years)		
Mean (SD)	45.4 (9.4)	49.7 (4.0)
Marital status		
Single	5	5
Cohabitant	11	17
Married	45	36
Divorced	13	13
Widow	1	1
Education ≤12 years	57 (76.0)	57 (79)
Employment status		
Employed	21 (28.0)	21 (29.2)
Not working/retired	1 (1.3)	1 (1.4)
Sick leave	17 (22.7)	20 (27.8)
Disability pension	36 (48.0)	29 (40.3)
Student		1 (1.4)
Duration of disease (years) Mean (SD)	7.03 (7.21)	6.13 (6.53)
Total	75	72

There were no statistical differences between baseline one and two in the intervention or waiting list group. The intra-class correlation (ICC) between Baseline 1 and 2 for GHQ20, ASES, EC-17 and FIQ varied from 0.66 to 0.84. Presented results are based on baseline two (three weeks before SMP) and post-test three weeks after SMP (Table 3).

**Table 3:** **Mean (95% CI) scores and treatment effect (differences between baseline and post-treatment, 3** weeks after intervention) *

	**SMP group**	**Control group**	**Treatment effect (95 CI)**	**P value**	**Effect size§**
***Primary outcomes:***					
**Psychological distress (GHQ20) (0 - 60, 0 =** **no distress)**				0.552	
Baseline	27.0 (11.0-57.2)	26.4 (10.0-50.2)			
Post-treatment	25.0 ( 6.0-49.1)	24.6 (10.0-57.2)	0.96 (-2.2 to 4.1)		0.10
**Effective Musculoskeletal Consumer Scale (EC-17) (0 -100 , 0** **= never, 100 =** **always)**				0.016	
Baseline	57.5 (22.1-88.2)	54.3 (0.0-86.8)			
Post-treatment	63.0 (36.8-97.1)	56.8 (1.5-100)	4.26 (0.8 to 7.7)		0.24
***Secondary outcomes:***					
**Self-efficacy pain (10 - 100, 100 = highSE)**				0.387	
Baseline	50.6 (18.0-82.0)	51.4 (10.0-98.0)			
Post-treatment	54.8 (16.0-94.0)	52.3 (10.0-82.0)	-1.83 (-6.0 to 2.3)		0.12
**Self-efficacy symptoms (10 - 100, 100 = highSE)**				0.189	
Baseline	57.8 (20.0-93.3)	57.7 (11.7-86.7)			
Post-treatment	61.4 (35.0-91.7)	57.9 (23.3-90.0)	2.63 (-1.3 to 6.6)		0.20
**Self –efficacy function (10-100, 100 = high SE)**				0.556	
Baseline	77.9 (22.2-100.0)	74.7 (18.9-100.0)			
Post-treatment	77.9 (32.2-100.0)	72.8 (16.7-100.0)	1.02 (-2.4 to 4.4)		0.06
**Fibromyalgia Impact Questionnaire (0-10, 0 = low impact)**				0.265	
Baseline	59.0 (16.1-89.6)	59.7 (23.9-92.5)			
Post-treatment	55.9 (7.0-90.5)	61.0 (23.2-93.2)	-2.76 (-7.7 to 2.1)		0.15

*Adjusted for gender, education, marital status and currently employed (Yes/No).

§Effect sizes were calculated between-group difference divided by the pooled SD of the baseline scores.

(<0.2 trivial; 0.2-0.49, small, 0.5-0.79, moderate, ≥80, large).

GHQ20, General Health Questionnaire, SMP, Self-management programme.

### Primary outcomes

No statistical differences were seen in the GHQ-20 between the groups (p = 0.55), except for a small statistically significant difference between the intervention and control group in the total score of the EC-17 three weeks after SMP, with a mean difference of 4.26 and SD 17.55 (p = 0.02) and an effect size of 0.24.

### Secondary outcomes

There were no significant differences in SEAS and FIQ between the intervention and control group three weeks after the SMP.

## Discussion

In this randomised controlled study, the main results showed no statistically significant differences between the intervention group and the waiting list group three weeks after completion of a one week SMP in psychological distress, functional and symptomatic consequences of FM and self-efficacy. There was, however, a small short-term beneficial effect on skills and behaviours which are important for effectively managing, participating in and leading one’s own health care (EC-17).

In our study, the participants had a high level of distress without achieving reduced level of distress three weeks after intervention. A review of cognitive-behavioural therapies and exercise programmes over 5-24 weeks for patients with fibromyalgia, reveals that participants who are characterized by high levels of distress seem to benefit most from non-pharmacological interventions [43]. Thus, the role of distress as a mediator of effect is warranted for further investigation.

In a qualitative study exploring participants' expectations prior to participation in the SMP, participants stated that they expected the SMP to empower them to take more responsibility for their own health and self-care [15]. These expectations are in line with the learning objectives of the SMP, which are to enable participants to be responsible and manage disease and daily life. The results in the present study, showing a small effect on coping skills and behaviours of an ‘effective consumer’, may indicate that participants are more aware of their own responsibility. This is important in a changing healthcare system that expects patients to be more active and responsible for managing their disease and healthcare [33].

Assessed on the basis of the didactic relations model [18,19], the lack of effect on distress, self-efficacy and functional consequences may have many explanations. It may be due to factors relevant to the participants' learning abilities such as age, education, motivation and the disease and its consequences. As the participants applied to take part in the SMP, it can, however, be assumed that they are motivated. Lack of effect may also be due to the content and contextual factors such as professionals' skills or the organisation of the programme [18,19]. Also, a one week programme may be too intensive or the content may not be in accordance with participants' needs. In other studies evaluating the effect of SMPs, the interventions are organised in shorter sessions over a longer period of time, which position the participants in a process where changes in knowledge, attitudes and behaviour can take place throughout the entire programme [8,44]. Teaching methods such as imparting information, counselling, exercises and exchange of experiences may contribute to the lack of effect [18,19]. In the qualitative study participants expected the SMP to facilitate acceptance, help them gain new knowledge and to be a forum in which to share their experiences. Participants who were employed assumed that participation in the SMP could help to ensure that they would continue in their jobs [15]. These are topics that are not currently prominent in the programme except the sharing of experiences. The didactic model also includes the choice of evaluation instruments and the suitability of the timing of the evaluation [18,19].

As far as we know, this is the first time the EC-17 is used as an outcome measure in the evaluation of a self-management intervention. The results confirm the findings in our previous validation study that the instrument can detect changes in the characteristics and skills necessary to be an active and participatory user of health services shortly after the intervention [35].

The present study has several limitations. One is that the participants were aware of their group allocation when they answered the questionnaires before the SMP (Baseline 2). However, there were no detectable differences within or between the groups at baseline 1 and 2, indicating that this knowledge had little impact on the completion of the questionnaires.

There are also limitations in the generalisability of the effect of this programme to other patients with FM. As the participants choose themselves to apply for participation in the SMP, they were also probably highly motivated to learn about how to cope with their illness and daily life.

Norway is a long and sparsely-populated country. Organizing the SMP as a one week intensive programme therefore makes it easier for patients who do not live close to the hospital to participate. Spending a whole week together can also give the participants more time for reflection, talks and discussions that can facilitate the learning process. The one week SMP should therefore be tested in a new study, which evaluates both the short term and long term (12 months or more) effects [12]. Before conducting a new study the programme should be reviewed on the basis of the didactic relations model of Hiim and Hippe [18,19], and additional topics described by participants in the qualitative study should be included [15].

## Conclusions

This study shows that in patients with FM, the SMP had no effect on psychological distress, functional and symptomatic consequences and self-efficacy, except for a small short-term effect on skills and behaviour that are important for managing and participating in health care (EC-17).

## Competing interests

The authors declare that they have no competing interests.

## Authors’ contributions

BH contributed to the study design, data collection, analysis and interpretation and writing of the manuscript; IK and KBH contributed to the study design, data analysis and interpretation and writing the manuscript; PM contributed to the data analysis and interpretation and writing of the manuscript. All authors approved the final version of the manuscript.

## Pre-publication history

The pre-publication history for this paper can be accessed here:

http://www.biomedcentral.com/1471-2474/13/189/prepub
